# Marte Meo as a port of entry to parental sensitivity - a three–case study

**DOI:** 10.1186/s12888-018-1959-5

**Published:** 2019-01-07

**Authors:** Elise H. Gill, Anne Brita Thorød, Kari Vik

**Affiliations:** 1 Sorlandet Hospital HF, Sørlandet Sykehus, ABUP, Serviceboks 416, 4604 Kristiansand, Norway; 2 Univeristy of Agder, Gimlemoen 25A, 4630 Kristiansand, Norway

**Keywords:** Marte Meo, Infants and toddlers, Parents experiences, Parental sensitivity, Attachment

## Abstract

**Background:**

Early parent- child relations play an important role in children’s development. Therapeutic intervention towards infants and toddlers at high-risk intends to prevent mental health problems. In this work, the parent-child-dyad is crucial. The video interaction guidance method, Marte Meo, is one among different methods used in attachment-based treatment in an outpatient infant mental health clinic. Parental sensitivity towards infants and toddlers needs is considered significant in developing secure attachment. Secure attachment is further considered decisive for mental health and the extent to which children are at risk of developing mental health problems. Different treatment methods aim at strengthening parents’ sensitivity. This study’s purpose was to gain further understanding about parent’s experiences with Marte Meo – therapy and highlight the importance for parental sensitivity.

**Methods:**

This is a cross-sectional phenomenological hermeneutical study. Four biological parents of three infants and toddlers aged 0–20 months who received Marte Meo- therapy in a clinical context were selected. Data was collected using semi structured interviews.

**Results:**

This article presents the study’s key-finding; we suggest that sensitivity increases. The key components of this are: watching edited video interaction in a therapeutic context, emotional activation, mutuality, self-esteem / self-confidence and reflective function. These are further elaborated and discussed.

**Conclusion:**

The findings indicate that Marte Meo contributes to facilitate development-supportive interaction, strengthen parental sensitivity, emotional availability, reflective functioning and coping - experience.

## Background

There are different methodological variations of video interaction guidance which are used in efforts to support the parent- child relationships [1–3]. Marte Meo is one of them [4]. This paper describes three cases from an outpatient mental health clinic at a hospital in Norway, in an infant – and toddler-team using an attachment-based treatment practice. The purpose of the study was to gain understanding of parent’s experiences with Marte Meo.

### Sensitivity and attachment

Winnicott [5] used the expression *there is no such thing as a baby, there is a baby and someone.* This can be viewed in a wider perspective. Gallese [6] detected the mirror neurons; their importance to intersubjectivity, the processing of actions of others, bodily sensations and emotions. Recent knowledge about epigenetic mechanisms highlights the interaction between genes and environment. Quality of care affects cognitive, emotional and behavioral development [7–9].

There is compliance between a parents working model of their child and the child’s attachment style. Children’s attachment-quality, working model of care and capacity to mentalize affect their capacity of emotional regulation. The child needs emotional co-regulation to develop the ability to self-regulate emotions. Emotional regulation is central for development of the Self [10]. Parents and children’s experiences and emotions are separate. In this context, Winnicott’s [5] *and,* in the expression *(…) a baby and someone* becomes even more meaningful*.* The ability to distinguish between *I* and *You* as separate is decisive in the parents’ reflective function.

Parental marked mirroring of the child’s emotional expression requires attunement. The child gets support in discovering themselves as separate from the other. The parents’ ability to mentalize and to be sensitive support the child’s development of self, reflective function, ability to mentalize, emotional regulation and attachment [11]. The term sensitivity in this context, is used both as such, as maternal and parental sensitivity [12, 13].

Ainsworth, Blehar, Waters, and Wall [12] defined maternal sensitivity as: *A mother’s ability to perceive her infants signals appropriately and respond to them promptly and adequately*. Nievar and Becker [14] included sensitivity, mutuality and synchronization in the term. Ainsworth described insensitivity where interaction initiatives and contact towards the child originate in the mother’s own wishes, impulses, mood and needs and not in response to the child [15]. Several factors affect parental sensitivity, e.g. the child’s temperament, socio-economical context-factors and parents working model of sensitivity and attachment, based on their own care experiences. Studies have supported Ainsworth’s hypotheses that parental sensitivity supports secure attachment [14, 16, 17], despite methodological challenges and variation in findings. Nievar and Becker [14] found that sensitivity was the most important parenting skill to promote secure attachment.

Attachment quality is one of the crucial factors for mental health of children and the extent to which they are at risk and vulnerable to develop mental health problems [18, 19]. Other risk factors increase this vulnerability. Early intervention towards infant and toddlers at high risk can prevent development of later emotional and social problems.

### Marte Meo

Marte Meo is Latin for *by my own strength.* Aarts and colleagues in the Netherlands developed the method in the − 80’s [4]. Aarts developed the method without any theoretical foundation. Some research on Marte Meo has been done, but there is need for more scientific knowledge. However, there are similarities with other methods which are more scientifically documented; attachment based methods like Video Feedback Intervention to Promote Positive Parenting (VIPP / VIPP- Relation / VIPP-Sensitive Discipline) [20] and psychoanalytically oriented-, systemic-, and transaction model-interventions [1, 2, 21, 22] . The methodology of Marte Meo has similarities with learning theory using positive reinforcement. Its approach is salutogenic [23]. It is resource, solution and empowerment focused. There are parallels with Bowlby’s [18] attachment theory, Sameroff’s [24] transaction theory, Stern’s [25] theories on early communication; intersubjectivity and attunement, and Fonagy’s theory of mentalization [26]. A part time two-year education consisting of theory and supervision on filmed Marte Meo-therapies leads to certification as a Marte Meo-therapist.

This method supports parents in developing supportive interaction with the child, while strengthening intersubjectivity and emotional mutuality. Aarts used the expression *Marte Meo is developing-time* [3]. Marte Meo is considered supportive of parental sensitivity [23, 27]. In our clinical practice, we use a more therapeutic approach than guiding.

The application of Marte Meo is such that every day- situations are videotaped. Hafstad and Ovreeide [28] coined the phrase *biopsy of lived life* for this. The therapist analyses which developmentally supportive elements of interaction present. There is focus on both incidence and quality of the interaction, atmosphere, emotional tone, sharing of emotions and joy. Stern’s [25] expression *moments of meeting* refer to moments of inter-subjectivity and intense sharing. Braten [29] articulated it as *felt immediacy.*

The parent receives feedback on the edited film about a week later. The therapist shows carefully selected scenes moment by moment with thoughtful use of still pictures. She is looking for what the child is already able to do, further their next stage of development and what the parent needs to discover to support this. Vik [23] coined the phrase *from the outside looking in.* Parents are watching the child and themselves from the outside on the film. The method supports an inside perspective on the child and an outside perspective on the parent themselves. The therapist tries to stimulate reflective dialogue. Parents’ descriptions of the child and their emotional expressions are important sources of information in the Marte Meo-therapy. The therapist’s sensitivity of the parents experience is crucial [30]. The exchange between the therapist, the parent and the image on the screen is significant. The therapist uses the developmental supportive elements of interaction in the therapeutic session as well. When required, the therapist makes new recordings. The therapy is considered complete when parents and therapist consider the challenges solved or when the developmentally supportive elements of interaction are present.

### Marte Meo and video interaction guidance in theory and research

Despite comprehensive searches in Pubmed and Psycinfo, little scientific work on Marte Meo was found. However, research suggests effect on parent-child-interaction. Marte Meo stimulates parents’ reflective functioning. It has a positive influence on parents’ recovery processes and reduction of depressive symptoms, increased vitality, strength, hope and improved self-esteem [3, 23, 30, 31].The therapist’s sensitivity and tuning- in is crucial. Vik [23] summarizes that what was working was to watch supportive interaction and mutual joy in the filmed dyads. The method supports the development of *new schemas of being together* [3].

Osterman, Møller, and Wirtberg [32] found the method suitable for supporting adoptive parents in giving their children developmental support. Bünder’s [33] study concluded that the method has greater impact on atmosphere than on structure, and that it supports communication, self-esteem and reflective function in parents. A Norwegian randomized controlled study (RCT) investigated a manual based version of Marte Meo. Video feedback was compared to treatment as usual in families with parent-child interaction problems. The study found evidence of a short-term effect especially among depressed parents, those with problematic interactions, and to some extent parents with personality disorders traits. They also found long-term effect on children’s social/emotional development and positive effect on parents’ depressive symptoms. The study support use of Marte Meo in families with interaction problems [34]. A recent study reports that the use of video-based education of health visitors increased their knowledge of, and skills in, assessing parent-infant interactions [35]. Kennedy et al. [2] found that Video Interaction Guidance (VIG) was effective in supporting development of secure attachment in their pre- and post- studies in a high-risk population. They found that focus on intersubjectivity was important. Further, they found that reflective function and mutuality in the relation are supported, self-esteem improves and stress reduces in parents. A tentative conclusion is that VIG can be very effective in supporting parent’s sensitivity and this can affect attachment security. Bovenschen et al. [36] studied the effect of video-feedback on sensitivity in a high-risk population. The method had significant effect by increased sensitivity measured short time after the intervention, but 6 months later, there was no difference compared to the control group. Doria et al.’s [22] study in another high-risk population concluded that Video Interaction Guidance (VIG) increases family joy, parental self-esteem and self-confidence. It changes their behavior because of key-elements in the method; therapeutic support, filmed interaction, solution focused approach and the film as a proof of success and change. The underlying mechanisms were metacognitive processes and construction of new meaning.

### Treatment intervention to support parental sensitivity and attachment

Many of the children who are patients in our clinic are at high risk due to their parent’s difficulties. Treatment of the mother’s mental illness does not necessarily improve interaction and mother-child-relation [21, 37]. The parent’s personal assessments are not always reliable in evaluating the quality of the interaction. This is why effort towards the relation and the child is crucial.

Early experiences with the caregiver affect development of attachment [12]. Bakermans-Kranenburg et al. [20] did a meta-analysis of experimental studies on sensitivity- and attachment interventions in early childhood, summarizing their findings by *less is more*. Therapy with a clear focus on sensitive care for a short period with intensive but few interventions has effect on both sensitivity and attachment security. Furthermore, they found most effect in those with insecure attachment-style, in high-risk relations, in clinical populations, and that interventions which involves fathers are more effective. They emphasize that interventions towards decreasing the psychosocial burden are necessary in addition.

Goodman [38] addresses different points of entry for attachment-based interventions. He distinguishes between two main levels. The first level is directed towards parents’ reflective functioning, their representations of care and of the child. The second level focuses on the relation with the child and their concrete behavior in the interaction. The main goal is to support the attachment- process. The sub goal is facilitating development-supportive interaction with the child, while also strengthening the parent’s sensitivity, emotional availability, experience of coping, and reflective functioning [26]. In attachment supportive Marte Meo-therapy it is especially important to focus on the child’s initiatives, experiences, emotional states and needs. Dialogue, rhythm and mutuality in the interaction are elements that we focus on. This supports attachment and development of the relationship [28]. Beebe [21] emphasizes the fact that viewing interaction from a distance supports mentalization and reorganization of representations. The Marte Meo method supports this; therefore, this study is needed.

## Methods

This is a qualitative three-case study with a phenomenological hermeneutical approach and in-depth interviews. Phenomenology is a research tradition that uses lived experiences and interpretation of meaning to understand a phenomenon [39].

### Sample

The sample was targeted. Two mothers and two fathers, all of them biological parents, representing three cases, participated as informants. They received Marte Meo in a clinical context. Two of them were a married couple. They had a newborn infant who was referred from the maternity ward. The mother was diagnosed with a severe depression. Another infant was referred from the family’s G.P. at the age of 6 weeks. The father suffered from anxiety and experienced difficulties in interacting with his child. The last infant was a few weeks old, referred from neonatal intensive- care unit because of his mother’s young age, her depressive symptoms and other psychosocial risk factors such as low income. The infants were all at high risk because of their parent’s difficulties. Their need for support in the attachment-process was the basis for the Marte Meo-intervention. The Marte Meo-therapy started immediately after referral. The informants received Marte Meo-therapy between two and eight times. After that, they participated in in-depth interviews. At the point of the interview, the children were four, nine and 20 months old.

### Data collection

The data was collected through in-depth interviews. A semi-structured interview-guide consisting of open-ended questions regarding the parents’ experiences with Marte Meo invited them to talk freely. The questions examined how it was to watch the filmed interaction, how they recognized the child needs, descriptions of the child and the relation. Further topics were the experience of being a parent and changes in these aspects through the therapy. The first author audiotaped and transcribed the interviews verbatim.

### Ethics

The 1964 Declaration of Helsinki and its later amendments guided the study’s ethics considerations and choices. The Regional Committee for Medical and Health Research Ethics (REK) in South- eastern Norway approved the study with reference number 2013/1335. The Norwegian Social Science Data Service (NSD) also approved this study, with reference number 35573. The Department for Child and Adolescent Mental Health and the Research Unit at the hospital gave their permission. Informed consent was obtained from all individual participants included in the study.

### Methodological considerations

The first author was both Marte Meo-therapist and researcher. This required thorough reflection on ethics and outcome [23, 40]. The study describes parents’ experiences qualitatively. The results are valid for these three cases. There are corresponding findings with other more comprehensive studies. This indicates extern validity. Interpretation and analysis have characteristics of social constructionism. My re-telling is my construction of reality, with my representations and theoretical pre-understanding. I have aimed at making evident my pre-understanding and every choice made in the research process. I have reflected upon them to contribute to transparence.

As a Marte Meo-therapist I have a positive pre-understanding of the method. The dual role as both therapist and researcher in all cases could lead to research bias. The study design would have been improved by having independent people leading the therapy, conducting the interviews and interpreting the results. Although, it is a goal that the research reflects the informants’ experiences; therefore, I have put myself in a non-judgmental position. This has implications for the study’s trustworthiness and validity of the study. Authors 2 and 3 contributed by looking at the material and the research process in a meta-perspective. Switching between therapeutic closeness and analytic distance are central in the study. The already established relation with the informant’s might have led to relational security, relaxed atmosphere, intersubjectivity and that it was easy to speak freely. This may have contributed positive to validity in data [41]. On the other hand, it could have led to an experience of being indebted that inhibited them from giving honest information. My pre-understanding could have influenced what I was able to understand and what I found in the experiences of the parents, therefore, theoretical in-depth knowledge was important.

### Analysis and interpretation process

The cases describe parents’ experiences with Marte Meo and shed light on how these affects parental sensitivity. Reflection and interpretation started during the interviews as data emerged. It was a dynamic process. Several interpretation styles or technics within qualitative research inspired the analysis. These include hermeneutical meaning-interpretation and meaning condensation described by Kvale [40] as well as theoretical reading described by Malterud [42]. The goal was to reach a deeper understanding where each part was seen in relation to the entirety. I switched between the text, my pre-understanding, theory and research. Sixteen meaning-units were condensed to the following:

- watching edited filmed interaction in a therapeutic context,
- emotional activation,
- mutuality,
- self-confidence/ self-esteem,
- reflective functioning/mentalization
- sensitivity

After further condensation and analysis of the process of the informant’s experiences, I found a dynamic pattern or a process towards the main finding of the three cases; increased sensitivity. A dynamic explanatory model presents the findings.

## Results

### To watch edited filmed interaction in a therapeutic context

*For my part it has been to watch and listen at the same time* (M1)Parents described that it was crucial for them to watch the film and at the same time listen to the sound of the baby’s and their own voice and the therapist’s guidance. Combination of modalities of senses makes it concrete and activating in several areas; perceptual and emotional. The images that are presented are described as emotionally activating. The video-technology allows for use of pause and slow motion. Oral guidance alone is susceptible to becoming abstract and general; while not providing the sense activation that is required. The parents described how the baby’s communicating skills became visible for them; therefore, increasing their attention to the situation.

“Things happen very fast and when one watches it on the film, one can stop slightly. That one sees things one has seen before, but it is more visible (…) that he communicates more than I thought (…) it has made me more attentive towards looking for minor things.” (F2)

Parents described instances where they discovered details about themselves, and how they were supported in noticing details of the child’s initiatives. They were supported in situational understanding and interpretation. The therapist change focus between the parent’s initiatives and what she considers moments of great importance. This implies sensitivity towards the parent’s signals, initiatives; expressions and emotions. The parents also described positive experiences about the use of micro-sequences in the film and the look!-effect. This supported meaning-construction and interpretation of the child. They described that details of the child’s expressions became more noticeable by pausing and showing sequences image by image. This created an authentic and genuine experience due to the strength of the visual images.

“ I have experienced it several times (…) about details, I think I am relatively good at seeing what he needs, but the micro- communication; such small things I have become more attentive to (…) it is no film-tricks either”. (M2)

The parents pointed out that it was important to watch themselves in interaction with their own child and that it would not have been the same to receive oral guidance without seeing with their own eyes simultaneously. *I have seen that I manage (…) it is not only that I can say It* (M1)*.* The parents described that the images were recorded to memory and reminded them of the child’s expression and dissemination in daily life. *It is such things that get stuck in my head* (M1). The informants did describe the film as a gift that gave them a positive experience; however it also became a reminder to be sensitive in day-to-day interactions.

### Emotional activation

Parents show various emotions when watching the film clips. Sometimes they laugh, sometimes they cry and sometimes they sit watching without expressing emotions*. I felt the tears pressing inside* (M1). Negative emotions: shame, guilt and frustration can be interrupted and more positive emotions can come through. One mother described the therapeutic feedback as a moment where she experienced new emotions. *Then I have glimpses of thoughts on that maybe the child does not experience it so badly* (M2). She described the experience of being able to do something good for her child, despite many difficult emotions about being a mother.

*I remember exactly the smile he gave me on the response I gave (…) it is such a thing that really has burned itself into my head* (F1). To show moments of joy to parents has huge emotional power. The experience of shared joy and delight are the most important moments to show. Fright and anxiety gave way to positive emotions for this father. He (F1) expressed that he experienced pleasure and new thoughts on how they interacted due to Marte Meo.

“I was so anxious (…) and I was so worried about the attachment (…) that she actually gave positive response and seemed to be feeling secure (…) and that I only with my voice could calm her”.

The picture he had of himself, the child and the dyad became filled with more positive emotions. To use images that represent a difference can change the perception that parents have of the child, themselves and the interaction. The parent can contribute to new stories about the relation. *It is yeah a part of reality. (…) It was an especially large gap between the film and how I myself thought it looked* (M2). For parents who are struggling with emotional connection with their child because of mental problems the method can contribute to new thoughts about themselves as mothers and fathers. The film becomes reality and their emotions can be affected by their mental condition; which is not reality.

### Mutuality

The informants emphasized the importance of the methodical focus on the relation and the mutual exchange that happened between parent and child. They described that the children’s increased vitality and bodily expression led to vitality and positive emotions for themselves as well. They experienced interaction happening, emotional exchange and moments of joy and intersubjectivity. One parent said that Marte Meo contributed to development, that it was a catalyst and a positive reinforcement. The next cited father (F1) emphasized the power of the child’s glance and his new interpretation of it when Marte Meo-therapy made him aware of it;

“What I earlier felt as coincidental glances at me, (…) now seemed as if she meant something about it and that she was seeking contact with me (…) it was not just by chance (…) I feel that when she is sad, I can answer in a way by the tone of my voice. I can show that I understand that she is sad (…). I feel that it has helped the way that I communicate with her, that I feel that I communicate at all”.

He described an experience of being able to share emotions with his child. He also described that watching the child on the film, and receiving Marte Meo-therapy, helped him feel participatory and important. He experienced mutuality and became more aware of his own communication.

### Self-confidence and self esteem

During this study, parents gained new insight into themselves and their parenting skills. They described an increased sense of security, care for themselves and sense of achievement. *It has made me much more self-confident (…) there are certain things I can do anyhow* (M2). Fear of not being good enough can preoccupy the parent and can prevent them from being communicating effectively with the child. For one father it sometimes made him insensitive to the child’s signals or led to negative interpretation. Marte Meo contributed to focus on the child’s signals and interaction moment by moment.

“What I earlier felt as coincidental glances at me, now seemed as if she meant something about it (…) it was not just by chance (…) I felt that it in a way did something good to me. (…) Then I relaxed so that I could rather focus on the positive things and her signals and have fun with her instead, rather than interpret everything as negative” (F1).

He described that less stress, improved self- esteem and higher self- confidence as a father gave him more room for joy and pleasure in the interaction. Another informant spoke about uncertainty he experienced in previous conversations and how Marte Meo felt like a vitamin injection. The informants articulated increased energy and vitality by what the child expressed in the film. This emphasizes the great power of visual and auditory impressions. By focusing on the child, relying on what they present, and following the initiatives it takes, Marte Meo has contributed to improved self-esteem and self- confidence. The film, coupled with therapeutic intervention can be a source of new insight, coping-experiences and empowerment. The therapist can support this by showing several images that support these new thoughts the parents have about themselves, the child and the dyad.

### Reflective functioning/mentalization

To use micro-sequences in the film to explore the child’s inner state; intentions, thoughts and emotions, activate parents exploration and curiosity regarding the child. It can support their reflective functioning or their ability to mentalize. The informants described that they reflected on the child’s expressions, experiences and inner state. They described being able to add perspective to their interactions, all while gaining insight into themselves; the child seen from the inside, and they themselves from the outside. Active use of the technical equipment is crucial; use of a pause-button, fast forward and rewind to explore the images. The parents emphasized the importance of time and space to reflect on what had been seen, heard and experienced.

“To take pauses in the film is incredible important, (…) to get one or two minutes to reflect on it. (…) You challenged me; why do you think he had that facial expression? What do you think he is thinking about? It is really quite positive to be challenged like that because one begins to reflect on what he expresses in different situations. (…) If I had not received Marte Meo, (…) I would not have been as reflected on things like his needs and emotions as I am now” (F2).

The therapist’s balance between strengthening and supporting positive behavior, and to challenge by asking reflective questions is crucial. This can be experienced as expectations of achievement, while not intentional, and requires therapeutic sensitivity. This father experienced it as appropriate and important. The impression is that it contributed to the experience of being competent as a parent. To be engaged in reflective dialogue stimulated him to look through the child’s eyes and to hold his mind in his. The child was seen with its own needs and emotions. He described the ability to look at the child’s condition from a meta- perspective. The informants emphasized that the visual and auditory impressions stayed in memory longer than the therapeutic session. It contributed to reflection in daily life even after the therapy.

### Increased sensitivity

The informants described the Marte Meo-therapy as a process where they became more attentive to details during communication with the child. They interpreted this in a more nuanced way and were more sensitive to the child’s needs during the interaction. One father (F1) described how he is attentive: he discovers, understands, interprets and adjusts to the child’s needs with timing.

“Yes, making her more secure and just showing her that I was there. Earlier it was just instinctive, it was just something one did, (…) that she actually could think that it was very scary to get that bodysuit over her head (…) in a way, just being able to talk to her with a pleasant voice. Then she became secure about that it is safe and dad is not going to hurt me. (…) It has made me very much more aware of that she actually want to communicate, and in one way or another try to interpret it. (…) It can make her calm; dad is here and he is trying to understand”.

What he described requires sensitivity towards the girl’s signals, mutuality, synchronization of movements and responses. He recognized that he both understood and met her needs. This experience gave him a good feeling*. (…) now I look at it as a signal of her emotions instead of just a coincident* (F1). New interpretation of the child as competent and intentional increased the attention towards the child’s signals. The father interpreted that the child’s behavior was not as coincidental once he received Marte Meo therapy. He felt important and acknowledged by his child, not by the therapist’s applause.

“In some situations he needs my behavior because no one else around him is as calm as I. (…) Then I quickly notices that if I speak calmly to him, it is very seldom that he cries when he is put to bed. (…) I do not know if I have become more aware of it or if I have done it all the time. (…) I might think that it has helped me, what to say; to avoid bigger outbursts sometimes” (M2).

This mother described an experience of calmness, capacity to discover, being able to understand, and an adjustment to the child’s needs. She described sensitivity as well as developmental support she was able to provide the child. Through her calmness and ability to tune in, the child may have experienced co-regulation; being understood and comforted. Previously, the mother expressed a feeling of emotional distance from her child. A father had the impression that he gave his daughter developmental support for emotional regulation and sensitive co-regulation. He got the impression that she felt understood and therefore wanted to communicate even more. *I can show that I understand that she is sad, it has led to (…) that she in a way want to communicate more and that she feels that we understand her* (F1)*.* The informants discovered the complexity of the children’s expressions through the Marte Meo-therapy. They are described as understandable in their signals and as easy to interpret.

“Facial expression, mimicking (…) that he in a way is active with his hands, he shows by his body –language that he finds it ok that I am present at that moment. (…) Details in how he reacts; facial expression and how he communicates, (…) I see it on him” (F2).

This summarizes that the father interpreted and tried to understand the child’s complex signals and expressions as communication. Parents described how they noticed expressions and needs in the films and that it contributed to recognizing this more frequently in daily interaction. Through micro-sequences and focus on moments in the films, they noticed how signals became more recognizable*. When we watched the video, (…) I interpreted her signals much better afterwards. I could see things that I had not noticed before* (F1)*.* The parents described that they developed. They learned more about themselves and their child.

“I have become more attentive to when he needs more concrete comfort and when it is enough to look at him and help him further. (…) The way he contacts me, he does it in more ways than what is obvious. (…) that he while he is busy with something else he also seeks contact, but it is in a way more subtle, as if just to see whether there is anybody there. (…) Very often he continues when he in a way has been seen” (M2).

A mother described how the discovery of her son’s contact-initiative was decisive for her fast response and also for him to feel that his needs were met. She thought she became more attentive to this through Marte Meo. She said that the child sometimes only needed to feel *seen*. Other times he needed active response from her to regulate emotions or states. She described how she adjusted her actions to the child’s expressions and needs. This mother reflected on the child’s inner state.

The study revealed that Marte Meo has strengthened parental sensitivity. Examples of this are presented in the model where the findings of dynamic interactions are depicted.

Quotations are marked with letters and numbers, Mother 1 = M1, etc. in parentheses.

## Discussion

**Fig. 1:**
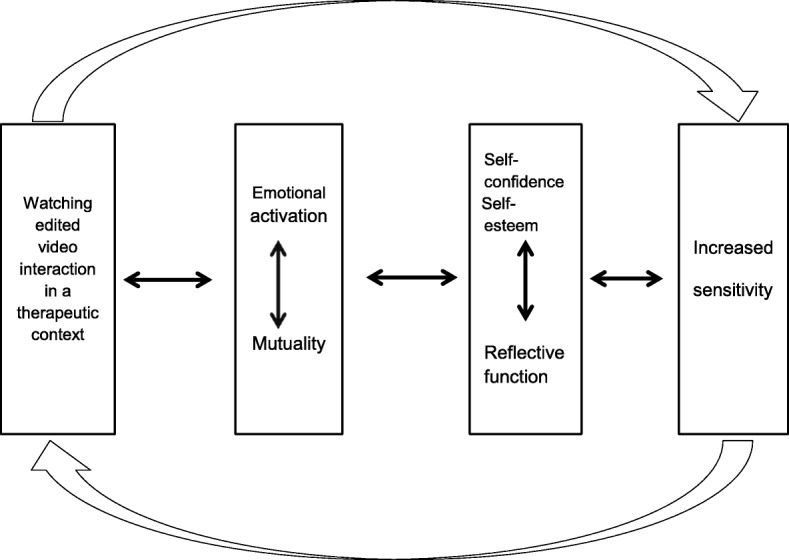
Dynamical explanatory model of parents experiences with Marte Meo

The parents’ descriptions shed light on how Marte Meo affects development of sensitivity and how it indirectly can impact the development of secure attachment. Essentially the discussion addresses the main finding of the study, but also the correlation in the explanatory model (Fig. 1). It presents parents experiences with Marte Meo and reflects my interpretation of the findings. The arrows of the model symbolize dynamics, interaction and the circularity in the therapeutic process. The model starts with watching edited filmed interaction in a therapeutic context. This activates the parents’ emotional response and supports the experience of mutuality in the interaction with the child. These factors work together. This also includes mutuality between therapist and parent. Parents experienced increased self-confidence, self-esteem, and reflective function. The main finding, increased sensitivity, is presented in the last window of the model. There are arrows backwards to show that there are interactions between the factors. The arrow back to the first window emphasize that increased sensitivity is seen as important in the next Marte Meo-session. This process can be repeated. The different factors in the model are each in their own way a catalyst for each other.

### To watch edited filmed interaction in a therapeutic context

The research question of this study differs from other studies on Marte Meo. Studies in non-clinical populations have nevertheless presented similar findings on key factors in the method. The informants conveyed that the use of film contributed to emotional activation that had the power to reveal and change emotions. Joy and delight in the interactions were strengthened. Further, the informants described that Marte Meo contributed to developmental-supportive interaction. This is in accordance with several of the aforementioned studies [3, 23, 30–32, 35].

### Emotional activation and mutuality

By applying a solution-focused approach with focus on developmental supportive interaction, new understandings, narratives, and perceptions were revealed. There can be parallels drawn to a study by Doria et al. [22] who found that new meaning was constructed. It seemed as if signals and mutuality had become more understandable as a consequence of perceptual and emotional activation through the therapeutic work. Despite possible unknown impact factors around the informants, they developed increased consciousness on the transactions in the interaction through the Marte Meo-therapy. The child as an active part in the interaction became visible to the parents. It received support in development of the self [5, 10]. The therapist’s sensitivity and intersubjectivity with the parents supports this development (Vik & Hafting, 2009). Kennedy et al. (2010) found the same about VIG.

### Self-esteem and self confidence

These parents described the experience of developing new thoughts about themselves through the power of images. Their self-esteem and self-confidence increased because of this. Likewise, parallels can be drawn to Bünder’s [33] Marte Meo-study and VIG-studies [2, 22]. Self-esteem and self-confidence provided space for increased calmness, delight, joy, and vitality in the interaction, which lead to increased sensitivity. This can also be seen in association with findings related to the effect on parental recovery-processes found by Vik [23]. The method can be assumed to be therapeutic to parents own behavior as well.

### Reflective functioning

The informants` descriptions can be understood by recognizing that a new perspective supported their reflective functioning [2, 21–23, 33]. The therapist’s reflective approach and the technological possibilities for still-pictures, slow motion etc., seem to have supported the parents’ reflective functioning, their ability to distinguish between the child’s and their own emotions. The findings emphasize that the Marte Meo-method supports mentalization and contributes to the distinction between *I* and *You* as the child’s own emotions become more visible [10, 23, 43]. As a result, the parents are supported in marked mirroring [43]. Furthermore, Marte Meo seems to contribute to the parents’ ability of co-regulation [10].

### Sensitivity

By comparing the informants` experiences of Marte Meo with how Ainsworth et al. [12] define sensitivity, and with Nievar and Becker’s [14] elaboration of the same term, we see parallels. The informants described having an increased sensitivity by improving their attentiveness towards the child’s signals and complex micro-communication. Further, they described that receiving Marte Meo-therapy helped them discover, understand, interpret and adjust to the child’s needs. They described synchronizing and timing of responses, and how the child as an active part in a mutual interaction was made evident and how that affected the parents’ response. Corresponding findings are reported by Kennedy et al. [2] in their VIG-study. They found the method effective in supporting parental sensitivity. The parents’ awareness of the child’s need for co-regulation by tuning- in and marked mirroring was possibly supported by Marte Meo [10].

### Marte Meo as a port of entry to parental sensitivity

Goodman [38] considers two different points of entry for attachment- based intervention. This study’s findings indicate that Marte Meo is directed against both of these points. It effects the first level by influencing the parents’ reflective functioning and the representations of the child, and at the second level by targeting relation and concrete interaction. Bakermans- Kranenburg et al. [20] consider interventions that support parental sensitivity and attachment to have effects especially in high- risk-relations, such as those with insecure attachment and in clinical populations. This indicates the usefulness of offering Marte Meo-therapy to dyads at high-risk as in this study.

Despite drawing from a small sample, this qualitative study has correlating findings with larger studies with quantitative design. This suggests external validity. There are RCT’s on various video interaction guidance- methods that indicate increased sensitivity in parents [13, 20, 34, 44]. Correlation between sensitivity and use of different video interaction guidance-methods are also found and emphasized by others [1, 2, 21, 36].

Nevertheless, it is not obvious that Marte Meo-therapy alone has contributed to the development of increased sensitivity. Several conditions could have affected the findings. The therapeutic relation can be an instrument that affects the informant’s experiences. My dual role as therapist and researcher could also have affected their answers in the study. The Marte Meo-therapy and the researcher-effect itself may have contributed to the parents` considering answers in a certain direction that would be more favourable. The informants are affected by other elements in their life as well; e.g. socio-economical context-factors and aspects of the child [45]. There can be several factors in their lives that the interviews do not reveal.

The findings of Bovenchen et al. [36] indicate that the effect of video interaction guidance can be time-limited. This indicates that follow-up Marte Meo-therapy should be considered. Everyday routine and the context of the informants’ life can contribute to less of a focus on interaction when the camera and film are turned off. The parents’ working model of care [10] and the support they get from their own social networks can affect how stable sensitivity is over time. Nevertheless, it seems as if parents’ working models of the child can be developed and changed through Marte Meo and its focus on positive reinforcement. Doria et al. [22] also found positive reinforcement and solution-focused approach as key- elements in VIG. As already mentioned, findings in this study indicate that parents get new ideas about themselves, the child and the dyad. Smith [45] considers this crucial to the child’s attachment-style. Children’s working model of care are supported by developmental supportive interaction. When parents increase their reflective functioning, children’s’ own reflective functioning is supported. Overall, this can be positive to the ability for emotional regulation and development of attachment. This study highlights that interaction-treatment using Marte Meo can contribute to development in especially vulnerable relations [21, 37].

## Conclusion

The study provides rich information about the method, its consistency with the therapy’s objectives and conceptual model. It highlights Marte Meo and the development of sensitivity. The findings illustrate the complexity of the experiences and how the key elements interact to increase sensitivity. To watch edited filmed interaction in a therapeutic context, emotional activation, experience of mutuality, increased self-confidence and self-esteem, as well as supported reflective function; all combine to contribute to development. The salutogenic approach of the method with resource-, solution,- and empowerment focus is considered as important in the process.

The main goal of treatment is to support the attachment-process. The findings indicate that Marte Meo contributes to the sub goal; facilitate development-supportive interaction, strengthen parental sensitivity, emotional availability, reflective functioning and coping experience. Despite the smallness of the sample, the study applies knowledge and understanding. The study supports use of Marte Meo in an attachment-based clinical praxis. This facilitates development of mental health. The study was conducted with parents of children at an age where attachment patterns are being established. They have received Marte Meo as support in the attachment-process. It is possible that the method should be combined with other attachment-based interventions if the children are older and if attachment- struggles already have occurred. As both therapist and researcher I may have contributed to research bias. Methodological triangulation would have strengthened the quality of the research. A quantitative approach e.g. using a pre-and post-intervention study or an RTC with sensitivity assessment would have contributed to the validation of actual effect. Outcome on actual attachment-quality could be measured by use of both pre-and post-study using Ainsworth’s Strange Situation Procedure (SSP) on children one year old and older both before and after the intervention. A longitudinal study would also be of interest. In this study there is no distinction between fathers and mothers. This could also be of interest in future studies.

## Abbreviations

E.G: Exemplia gratia/for example
ETC: Etcetera
